# Prevalence and risk factors of frailty in people experiencing homelessness: A systematic review and meta-analysis

**DOI:** 10.1016/j.tjfa.2025.100029

**Published:** 2025-03-04

**Authors:** Thomas Cronin, David Healy, Noel McCarthy, Susan M Smith, John Travers

**Affiliations:** a Irish College of General Practitioners / Discipline of Public Health and Primary Care, School of Medicine, Trinity College Dublin, Dublin, Ireland; b GP, Dublin, Ireland; c Discipline of Public Health and Primary Care, School of Medicine, Trinity College Dublin, Dublin, Ireland

**Keywords:** People experiencing homelessness, Frailty, Prevalence, Risk factors

## Abstract

**Background:**

The experience of homelessness has been associated with premature ageing and an earlier onset of geriatric syndromes. Identification of frailty and appropriate intervention, may help improve health outcomes for people experiencing homelessness (PEH). This review aimed to identify prevalence, use of screening tools and risk factors for frailty in PEH.

**Method:**

A systematic review, conducted and reported following the PRISMA checklist, was undertaken investigating the prevalence and risk factors of frailty among PEH. Searches were conducted in Ovid MEDLINE, PsycInfo, Web of Science and CINAHL from inception to July 2024. A meta-analysis examining prevalence of frailty and pre-frailty was completed with a narrative synthesis of related risk factors.

**Results:**

A total of 1672 articles were screened for eligibility and 11 studies were included, containing 1017 participants from seven countries. Six different screening tools were employed to detect frailty in the included studies. The range of frailty prevalence was 16–70 % and pre-frailty prevalence was 18–60 %. The pooled frailty prevalence from studies employing the Fried Criteria was 39 % (95 % CI 15–66); the Clinical Frailty Scale: 37 % (95 % CI 24–51); the Edmonton Frailty Scale: 53 % (95 % CI 44–63); and the Tilburg Fraily Indicator: 31 % (95 % CI 8–60). High heterogeneity was observed between the studies. Identified risk factors for developing frailty in PEH included being female, increased years spent homeless, and drug addiction.

**Conclusion:**

This study highlights a high prevalence of frailty and pre-frailty in PEH. The identified risk factors illustrate potential areas to target interventions to reverse frailty. Future research should focus on the role of screening for frailty in PEH and developing appropriate frailty detection tools in this group.

## 1 Introduction

Homelessness encompasses various living situations, including rough sleeping, emergency accommodation, and insecure housing. Whilst no universal definition exists, it is widely acknowledged that homelessness extends beyond the lack of physical shelter [1,2].

Globally, the number of PEH is increasing [3], with the average age of PEH also rising [4]. Structural, societal, economic factors – such as poverty, housing unaffordability, unemployment and discrimination – drive homelessness [5], alongside unmet health and social care needs, which can both contribute to as well as result from their homelessness [6].

### 1.1 Homelessness and health

PEH encounter significantly poorer health outcomes and reduced life expectancy [7,8]. Mortality rates are eight times higher than the general population for men and twelve times higher for women [9]. Previous research has indicated many of these premature deaths are from preventable and treatable conditions, linked to factors including psychological trauma, drug misuse and social exclusion [[10], [11], [12]].

It has also been recognised that PEH present with a variety of geriatric syndromes (e.g., functional limitations, falls, cognitive impairment, and urinary incontinence) at a younger age compared to the housed population [13,14]. In addition, PEH use more acute hospital services and emergency care than the general population, as well as the length of hospital stay being longer due to multiple needs [15,16]. As a result, it is suggested PEH are susceptible to premature ageing, whereby a person's physiological health and biological age deteriorate earlier and/or more quickly compared to other people of a similar chronological age [14]. Furthermore, PEH are considered more vulnerable to disproportionate changes in health status following relatively minor stressor events [17].

### 1.2 Frailty and homelessness

Frailty is regarded as an evolving concept and is defined as a “*state of vulnerability to poor resolution of homoeostasis after a stressor event and is a consequence of cumulative decline in many physiological systems during a lifetime*” [18].

A range of validated measures are available to assess frailty based on different models, and can help predict adverse outcomes and identify those who require additional support [19]. Feasible interventions have been shown to reverse frailty in older adults in the primary care setting [[20], [21], [22]].

Frailty may represent a key construct for understanding health vulnerabilities faced PEH because it can encapsulate the cumulative impact of physiological, psychological, and social stressors unique to this population [23]. The Frailty Framework among Vulnerable Populations (FFVP) emphasises how intersecting individual-level (e.g., chronological age), situational (e.g., first time homeless), health-related (e.g., comorbid conditions), behavioural (e.g., drug and alcohol use), and resource (e.g., social support) risk factors contribute to frailty onset in underserved groups such as PEH [24,25]. This framework highlights the need for tailored interventions that address not just physical decline but also the broader social determinants of health affecting PEH [26].

To help inform appropriate interventions and policy development in this area, it is important to better characterise the concept of frailty in PEH and establish the scale of frailty in this group. As such, this study aimed to determine the prevalence of frailty in PEH, explore the frailty screening tools used and examine the risk factors that influence the likelihood of developing frailty in PEH.

## 2 Methods

The protocol for this study was registered with the PROSPERO database (CRD42024519685) and we report our work using the PRISMA guidelines (Supplementary Material Section 1) [27]. The described methods below differed slightly from the protocol as the STATA 18 statistical software package [28] was utilised rather than MetaXL, as reported in the protocol. This was due to the former having a specific meta-analysis function for pooled prevalence.

### 2.1 Inclusion criteria

The inclusion criteria were based on recommendations by the Joanna Briggs Institute (JBI) for systematic reviews of prevalence [29], which is based on the CoCoPop mnemonic (Condition, Context and Population) and for aetiology and risk [30], which is based on PEO (Population, Exposure of interest, Outcome) (Table 1). Individuals over 18 years were included to reflect the demographics of PEH, given the concept of premature ageing in this group and that frailty may occur at any age [14,24,31]. Different legal definitions of homelessness are used amongst countries making consistent identification of people who are homeless problematic, whilst academic study may employ differing criteria for defining homeless [32]. As such, this review utilised the ETHOS typology to provide a framework to identify and categorise homelessness within the studies [1].

**Table 1:** Inclusion criteria.

*Population and Context*	*Condition and Outcome*	*Exposure of interest*
Persons over 18 years experiencing homelessness worldwide. This was informed by the FEANTSA (European Federation of organisations working with the people who are homeless) definitions of “roofless” (e.g. no fixed abode, living in a public space) and “houseless” (e.g. living in hostel, refuge, shelter, temporary accommodation) [1].	Studies reporting on frailty prevalence and incidence, with frailty assessed through a recognised measure for the detection of the frailty syndrome on the basis of a 2019 systematic review identifying 51 separate frailty measures [19].	Studies reporting on risk factors for developing frailty in people experiencing homelessness, consisting of biological, lifestyle, psychological and social factors.

Peer-reviewed studies of any design that reported original research on frailty prevalence and risk factors in PEH were deemed eligible for inclusion. Grey literature was not included, based on workload capacity of the team and to maintain methodological consistency and ensure the reliability of the evidence included.

### 2.2 Search strategy

A search strategy involving searches on Ovid MEDLINE, PsycInfo, Web of Science, and CINAHL (Supplementary Material Section 2) in consultation with a medical librarian was developed. Human limits were applied to the searches to filter the results to studies that involve human participants and exclude animal studies and in vitro research. There were no language limits and databases were searched from inception until the time searches were undertaken in July 2024.

Hand searches were also undertaken by screening the reference list of articles included in the systematic review. Additionally, for articles deemed eligible for inclusion, the Google Scholar ‘cited by’ feature was employed to identify articles that had cited these included works.

### 2.3 Study selection

The identified citations were imported into EndNote Online and duplicates were removed. Two reviewers (TC and DH) performed title and abstract screening and full-text review. Where differences in selection occurred, this was resolved through discussion, and involved a third reviewer (JT or SMS) if necessary. Authors of potentially included papers were contacted if points of clarification were needed.

### 2.4 Assessment of quality

The methodological quality of the studies was assessed by two reviewers (TC and DH). Reviewers used the JBI Critical Appraisal Checklist for Studies Reporting Prevalence Data [29] and the JBI Critical appraisal checklist for analytical cross-sectional studies [33], where risk factors were examined, and a quality score was assigned.

### 2.5 Data extraction

Data were extracted for included studies by one reviewer (TC) on the following: first author and year of publication; geographic location; study type; study aim; recruitment strategy / exclusion criteria; sample size; participants’ characteristics (age and gender); frailty measure used; frailty prevalence; identified risk factors for frailty in the population if applicable and divided into biological, lifestyle, psychological and social factors. The data extraction table was cross-checked by a separate reviewer (DH).

### 2.6 Data synthesis

Meta-analysis was conducted using STATA 18 meta tools to derive the pooled estimation of prevalence for frailty and pre-frailty from the included studies where this was reported. As frailty models are not considered interchangeable [34], each measure was assessed separately. For frailty prevalence, meta-analysis was performed for the following frailty models: Fried Criteria, Clinical Frailty Scale, Edmonton Frail Scale and Tilburg Frailty Index, and for pre-frailty prevalence for the following models: Fried Criteria, Clinical Frailty Scale, and Edmonton Frail Scale. The Freeman-Tukey Double Arcsine Transformation was used to stabilise the variances. A restricted maximum likelihood random effects model was fitted to account for the variability between studies in their designs and sizes [35]. Heterogeneity was assessed by using the *I*^2^ statistic, with *I*^2^ values of 25 %, 50 % and 75 % being considered to indicate low, moderate and high heterogeneity, respectively [36]. The proportions with their 95 % confidence interval values from individual studies were also presented in a forest plot. Due to the limited number of studies reporting on risk factors for frailty in PEH, this was narratively synthesised as informed by Popay and colleague's guidelines [37].

Publication bias was assessed by visual inspection of a funnel plot using the prevalence of frailty and an Egger test for statistical significance.

## 3 Results

### 3.1 Search results

**Fig. 1:**
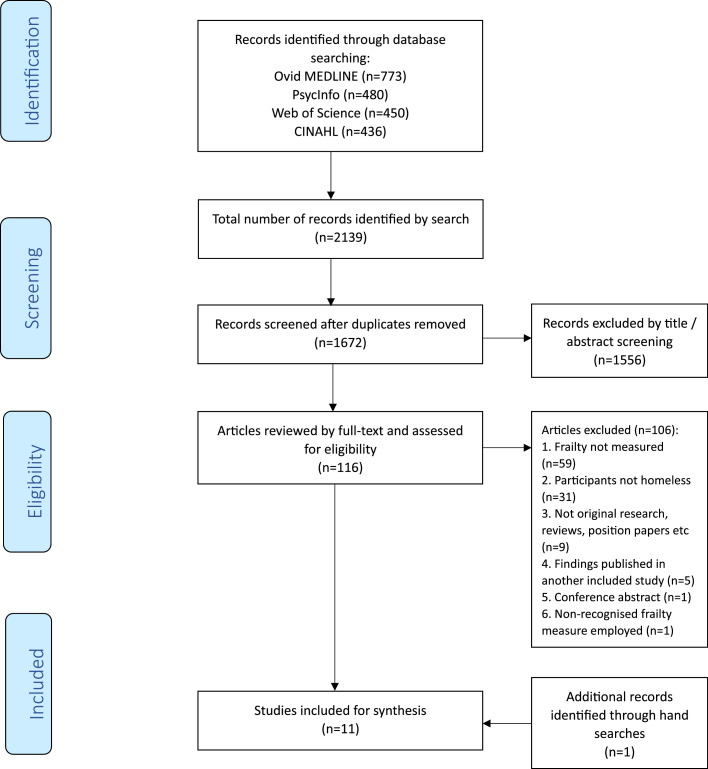
PRISMA Flow Diagram.

A total of 1672 articles were reviewed by title and abstract screening and 116 were selected for full-text review. Reasons for studies to be excluded after full-text review included frailty not being measured in the study, the population not identified as being homeless and non-original research. One study was identified from the ‘Cited by’ feature on Google Scholar. A total of eleven studies were ultimately included in the systematic review. Ten of the 11 studies reported on the prevalence of frailty and six of these ten studies reported on frailty and pre-frailty prevalence. Four of the 11 studies contained data pertaining to risk factors for frailty in PEH. Fig. 1 outlines the process by which these were chosen. **Study characteristics**

Characteristics of the studies and study samples are summarised in Table 2 and further detailed in Supplementary Material Section 3 as per the data extraction plan. The eleven included studies were published between 2013 and 2024 and were all in the English language. All studies took place in either the USA (*n* = 5) or European countries (*n* = 6) and involved a total of 1017 participants who had frailty assessed. For the ten studies that examined frailty prevalence, six were cross-sectional in design, two were feasibility studies, one was a single-arm trial and one was a case-control study. The four studies that assessed risk factors for frailty were all cross-sectional in design. A total of six different frailty measures were used in studies to identify frailty, with two studies using more than one frailty measure. The most frequent were the Fried Criteria (*n* = 4, 33.3 %), Tilburg Frailty Index (*n* = 3, 25 %) and the Clinical Frailty Scale (*n* = 3, 25 %).

**Table 2:** Study characteristics and sample characteristics of the included studies (*n* = 11) in people experiencing homelessness.

Study first author and year	Country	Study type	Study setting	Recruitment strategy	Sample size	Average age (years) of the total sample*	Female (*n*=, %)	Frailty measure used	Frailty prevalence (*n*=, x%)	Pre-Frailty prevalence (*n*=, %)
Brown 2013 [38]	USA	Cross-sectional	Day shelters, Homeless hostels	Systematic random	250	Mean=56 SD=5.3 Range 50–85	*n* = 48, 19 %	Fried Criteria	*n* = 40, 16 %	Not available
Salem 2013 [39]	USA	Cross-sectional	Day shelters, Homeless hostels	Voluntary response	150	Mean=52 SD=6.8 Range=40–73	*n* = 75, 50 %	Frailty Index	*n* = 81, 54 %	Not available
Salem 2019 [40]	USA	Cross-sectional	Day shelters	Voluntary response	130	Mean=39 SD=11.4 Range=19–64	*n* = 130, 100 %	Tilburg Frailty Index	Not available	Not available
Rogans-Watson 2020 [41]	UK	Cross-sectional	Homeless hostel	Convenience	33	Mean=56 SD=10 Range=38–74	*n* = 3, 9 %	• Fried Criteria • Clinical Frailty Scale • Edmonton Frail Scale	• *n* = 18, 55 % • *n* = 16, 48 % • *n* = 18, 55 %	• *n* = 13, 39 % • *n* = 11, 33 % • *n* = 6, 18 %
Kiernan 2021 [42]	Ireland	Cross-sectional	Hospital inpatients classified as homeless on admission	Convenience	63	Median=45 IQR=38–56 Range=23–80	*n* = 21, 33 %	Clinical Frailty Scale	*n* = 25, 40 %	*n* = 18, 29 %
Nyamathi 2022 [43]	USA	Single-arm trial	Day shelters, Homeless hostel	Voluntary response	50	Mean=54 SD=12.1 Range=27–76	*n* = 13, 26 %	Tilburg Frailty Index	*n* = 23, 46 %	Not available
Lowrie 2023 [44]	UK	Cross-sectional	Day shelters, Homeless hostels	Purposive	71	Not available	Not available	Fried Criteria	*n* = 50, 70 %	*n* = 20, 28 %
Speck 2023 [45]	Germany	Cross-sectional	Primary care clinic for people experiencing homelessness	Convenience	60	Median=48 IQR=39–58, Range=not available	*n* = 7, 12 %	Fried Criteria	*n* = 13, 22 %	*n* = 36, 60 %
Nyamathi 2023 [46]	USA	Case-control	Homeless hostels, Drug treatment centres	Convenience	105	Mean=45 SD=not available Range=18–77	Not available	Tilburg Frailty Index	*n* = 19, 18 %	Not available
Shulman 2024 [47]	UK	Feasibility	Homeless hostels	Convenience	74	Mean=49 SD=not available Range=22–82	*n* = 20, 27 %	Edmonton Frail Scale	*n* = 39, 53 %	*n* = 17, 23 %
Kennedy 2024 [48]	Ireland	Feasibility	Day shelter	Voluntary response	31	Mean=45 SD=16 Range=22–77	*n* = 11, 35 %	• Clinical Frailty Scale • SHARE-FI	• *n* = 7, 23 % • *n* = 5, 16 %	• *n* = 8, 26 % • *n* = 6, 19 %

⁎ For the average age column, eight studies reported mean age, two studies reported median age and one study did not have this available. Where reported, standard deviation (SD) for mean and interquartile range (IQR) for median and age range are also shown.

### 3.2 Quality assessment and publication bias

The ten studies that reported frailty and pre-frailty prevalence data were assessed using the JBI Critical Appraisal Checklist for Studies Reporting Prevalence Data (Supplementary Material Section 4). All ten of the studies had a quality score above 50 % and the mean quality score was 72.5 % (9.3 %, SD). In addition, four of the studies were assessed using the JBI Critical appraisal checklist for analytical cross-sectional studies (Supplementary Material Section 5), and the mean quality score was 62.5 % (12.5 %, SD).

**Fig. 2:**
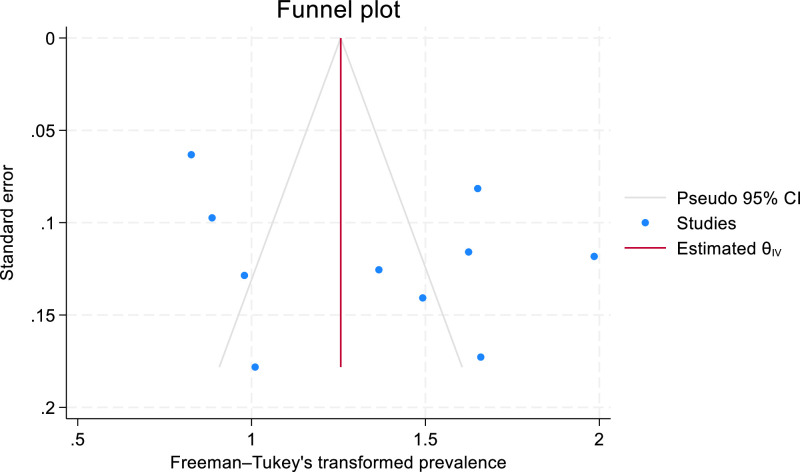
Funnel plot of the ten studies reporting prevalence data on frailty in people experiencing homelessness.

Publication bias was assessed based on the prevalence of frailty reported in ten of the included studies. Where studies used more than one frailty measure (*n* = 2), the value from the most frequent frailty measure in the systematic review was used, to reflect the measure most frequently applied in the literature and offer a balanced comparison. The funnel plot (Fig. 2) revealed a broadly symmetrical distribution of the studies suggesting publication bias was not present, whilst Egger's test indicated no small study effects (*p* = 0.58).

### 3.3 Prevalence of frailty and pre-frailty in people experiencing homelessness

In the ten studies reporting frailty prevalence the range was 16–70 % and in six studies reporting pre-frailty prevalence the range was 18–60 %.

**Fig. 3:**
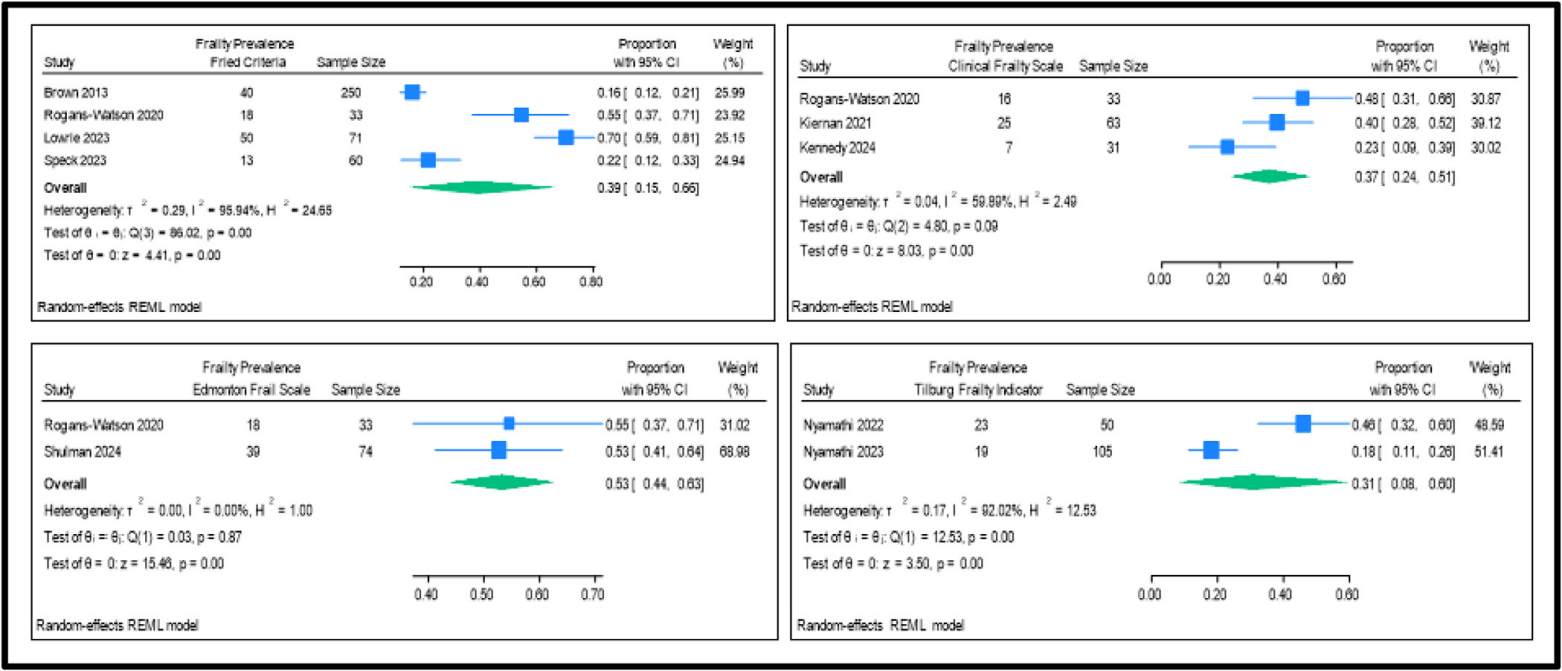
Pooled prevalence of frailty, as measured by frailty tools that were reported more than once in the included studies.

The pooled frailty prevalence from the four studies employing the Fried Criteria was 39 % (95 % CI 15–66), with an *I*^2^ of 95 % indicating high heterogeneity (Fig. 3). For the Clinical Frailty Scale, reported in three studies, it was 37 % (95 % CI 24–51), with an *I*^2^ of 60 % indicating moderate heterogeniety. For the Edmonton Frailty Scale reported in two studies, it was 53 % (95 % CI 44–63), with an *I*^2^ of 0 indicating no heterogeneity. Lastly, the Tilburg Fraily Indicator was reported in two studies and pooled prevalence was 31 % (95 % CI 8–60), with an *I*^2^ of 92 %, indicating high heterogeneity.

**Fig. 4:**
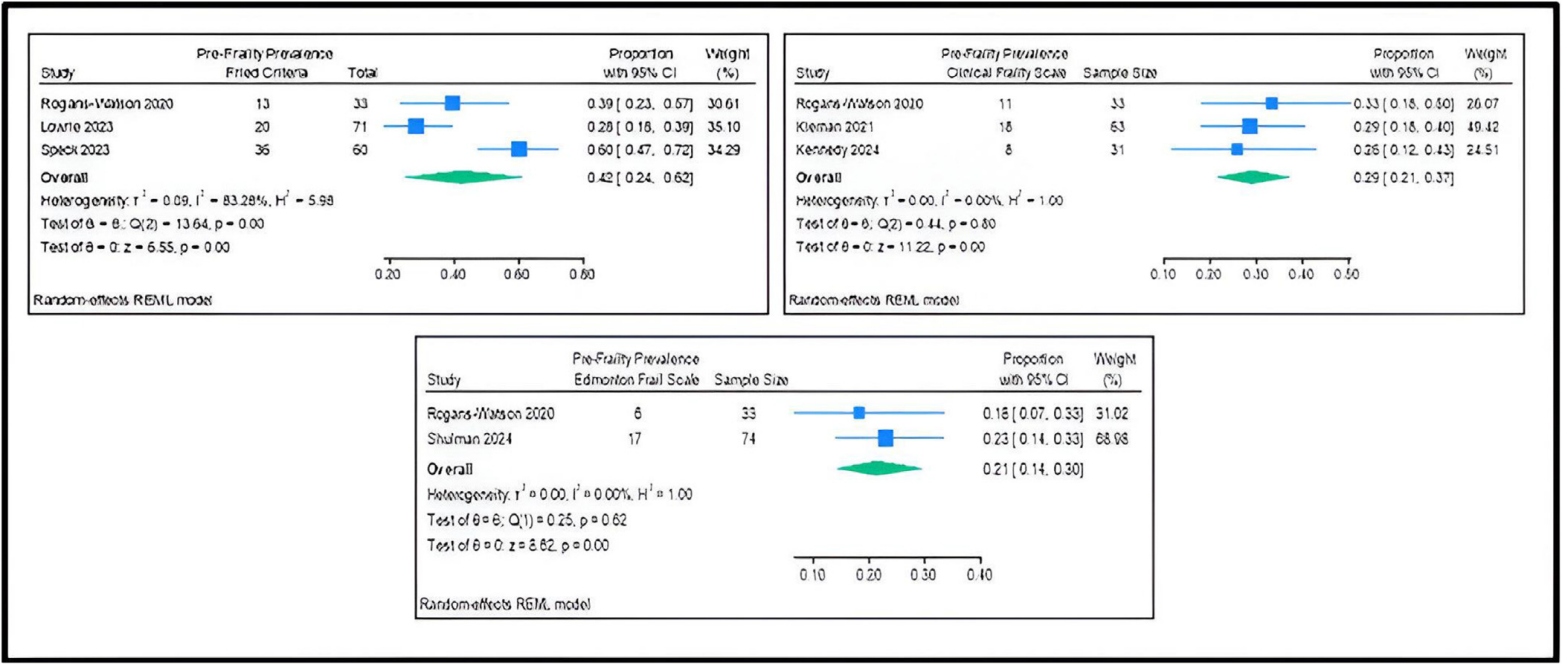
Pooled prevalence of pre-frailty, as measured by frailty tools that were reported more than once in the included studies.

The pooled pre-frailty prevalence from the three studies employing the Fried Criteria was 42 % (95 % CI 24–62), with an *I*^2^ of 83.2 % indicating high heterogeniety (Fig. 4). For the Clinical Frailty Scale, reported in two studies, it was 29 % (95 % CI 21–37), with an *I*^2^ of 0 indicating no heterogeniety. Lastly, for the Edmonton Frail Scale, reported in two studies, it was 21 % (95 % 14–30), with an *I*^2^ of 0 indicating no heterogeniety.

### 3.4 Risk factors for frailty in people experiencing homelessness

#### 3.4.1 Biological and psychological

Biological and psychological risk factors associated with frailty in PEH were reported in four studies. One study reported that of the participants recorded as having frailty, 95 % (*n* = 38) had >1 other geriatric syndrome, 88 % (*n* = 35) had >2 other geriatric syndromes, and 38 % (*n* = 15) had >4 other geriatric syndromes [38]. Another study reported an association between frailty and lower nutritional status [39]. A third study reported that higher post-traumatic stress disorder symptom scores were associated with higher levels of physical frailty (*p* < 0.05) [40].

Two studies also reported statistically significant higher frailty levels in females compared to males in PEH [39,42]. Mixed results were identified around the association of age and frailty. One study reported that those who were older had higher physical frailty scores (*p* < 0.01), but lower levels of psychological frailty (*p* < 0.05) [40]. Meanwhile, another study using stepwise regression found that increased age was significantly associated with being frail in the studied population [39]. A further study examining the association with age did not find any significant difference in frailty scores between age groups (*p* > 0.05) [42].

#### 3.4.2 Social and lifestyle

Two studies reported on social and lifestyle risk factors. One study demonstrated a positive relationship between healthcare usage and frailty in PEH [39]. In addition, another study found that those who were homeless for a higher number of years had higher physical and psychological frailty scores (*p* < 0.05) [40]. This study also found that those who had a higher drug dependence had greater physical, psychological and social frailty scores (*p* < 0.05).

## 4 Discussion

### 4.1 Principal findings

This systematic review and meta-analysis synthesised data from eleven studies that included a total of 1017 adults experiencing homelessness who had their frailty status measured. Frailty was assessed using a range of different tools and a variety of different study methodologies were included. Nevertheless, across all measures, frailty and pre-frailty were highly prevalent amongst PEH, with a pooled prevalence of the frailty tools ranging from 31 to 51 % and 21–42 %, respectively. High levels of heterogeneity were observed, as well as a wide variance in frailty and pre-frailty measures and in prevalence detected. This may be related to measures being reported in studies undertaking different recruitment strategies, targeting different populations amongst PEH and varying exclusion criteria. For instance, some studies recruited from a hospital / clinic setting [42,45], whilst others recruited from homeless hostels and shelters [40,49]. Furthermore, some studies either had predominantly male [41] or exclusively female populations [40].

### 4.2 Comparison to existing literature

Whilst estimates for frailty prevalence in the population can vary depending on the setting [31,[50], [51], [52], [53]], these levels in PEH appear to be higher than the reported pooled prevalence of 10.7 % (95 % CI 10.5–10.9) in community dwelling adults aged over 65 [54], with frailty in PEH often multiples of this, and closely resembling the pooled prevalence of 52.3 % (95 % CI 37.9–66.5) of those identified in nursing homes [55]. This review is consistent with evidence demonstrating a higher frailty prevalence among people with a lower socioeconomic position and that frailty also occurs earlier and progresses more rapidly in those experiencing socioeconomic deprivation [[56], [57], [58]].

Only four of the 11 included studies examined risk factors for frailty in PEH. Being female was associated with frailty in PEH, and this is consistent with existing literature in the field that has identified clear sex differences in frailty, with females almost always having higher frailty prevalence [54,59,60]. Drug dependence was also linked to frailty, which has been identified in other research [61,62].

Frailty and multimorbidity have been identified as associated conditions and they often overlap, particularly in older people [63,64]. This review has identified high levels of frailty in PEH and high levels of multimorbidity have also been reported previously in PEH [[65], [66], [67]]. Our review highlights that the number of years experiencing homelessness was associated with frailty development, which is consistent with the more intense burden of ill-health in chronically homeless adults [68]. The co-existence of frailty and multimorbidity is also associated with increased mortality risk, even after adjusting for the number of conditions, sociodemographic variables and lifestyle [69]. Socioeconomic deprivation appears to play an important role in the development of both frailty and multimorbidity [58,70].

Negative social determinants of health including smoking, social isolation, sleep disturbance and adverse childhood experiences (ACE) are found at higher levels in PEH in contrast to the general population [[71], [72], [73], [74]], and are also factors associated with the presence of frailty [61,[75], [76], [77]]. These may represent key drivers for the higher rates of frailty observed PEH, which may impact upon the different ‘hallmarks’ of biological ageing, such as telomere attrition and cellular senescence [78].

It is also recognised that frailty in older people contributes to higher health service usage [79]. Similarly, being homeless has been associated with recurrent emergency department attendances, high hospital readmission rates and longer hospital stays, compared to the general population [15,16]. This highlights the increased complexities and cost of health treatment for PEH, particularly when compounded by the high levels frailty and pre-frailty they experience as demonstrated by this review. Moreover, this review highlights how risk factors for frailty in this group including drug dependence and chronic homelessness can make care for PEH more challenging, since these factors can create barriers to receiving quality medical treatment, and complicate adherence of recommendations from healthcare providers [14].

In regard to the identification of frailty, numerous methods exist, incorporating different constructs, and developed for different purposes [19]. Previous research has indicated that there can be different rates for frailty depending on the tool used [[80], [81], [82]]. In addition, certain frailty instruments may contain distinct constructs not contained in other instruments. For instance, the Tilburg Frailty Index involves a social parameter [83], whilst the Fried Criteria is mainly focused on physical domains [84]. As such, it is recognised that frailty identification methods are not necessarily interchangeable [34].

Furthermore, with relevance to our population, many frailty tools have not been validated for use in younger populations (typically below 60 years). Indeed, there are currently no validated tools to identify frailty exclusively in younger groups [85]. Therefore, there may be issues within the included studies around the validity of the identification of frailty. However, it is worth noting that two of the included studies used more than one frailty measure, and prevalences of frailty remained consistent.

### 4.3 Strength and limitations

The review employed a comprehensive search strategy, combining electronic and hand searching, with no date or language restrictions. It employed more than one reviewer in the independent selection, quality assessment and data extraction process improving the reliability of its findings.

The Grading of Recommendations, Assessment, Development, and Evaluations (GRADE) was not performed due to capacity constraints [86]. However, the study designs, generally small sample sizes and high heterogeneity in many of the findings suggests lack of certainty and a need for some caution in interpreting the findings. In addition, grey literature was not searched, and its inclusion may have captured a broader range of insights and led to some publication bias in our results, though our assessment of publication bias does not suggest it was an issue.

A further limitation was lack of a grey literature search, based on team capacity and project timelines. This may have led to some publication bias in the current review, though our assessment of publication bias does not suggest it was an issue. However, expanding the search was beyond the workload capacity of the research team and the methodologically rigorous studies within our focus were expected to be submitted for peer reviewed publication.

This study adopted the FEANSTA classification of “roofless” and “houseless” [1]. The “houseless” part of this definition can include people living in women's shelters to those due to be released from institutions. Whilst this review included studies that recruited participants from a variety of settings, not all types of these categories were represented. It may be that varying rates of frailty would be identified amongst these different settings. Additionally, individual level data was not available to be extracted in the included studies regarding the characteristics of those who were frail compared to non-frail, with respect to demographics and homeless category, preventing subgroup analysis by age and homeless situation. This may have produced findings to inform policy and practice more meaningfully.

Lastly, for many of the studies, the primary objective was not necessarily to determine the prevalence of frailty in PEH. Therefore, recruitment strategies were often tailored for other reasons and could introduce bias into the rates of frailty prevalence measured. Nevertheless, given that PEH are frequently neglected in research, and the idea of premature ageing in PEH is a relatively new concept, it may be anticipated this is not a widely studied phenomenon and collating the existing research in this field is useful endeavour to better highlight the issue.

### 4.4 Implications for policy, practice and future research

This review highlights high levels of frailty in PEH illustrating a pressing need for policy changes to address issues that contribute to homelessness. This further adds to the evidence base of the adverse health effects associated with homelessness. Risk factors for frailty in this group include drug addiction and increased time spent homeless emphasising the prioritisation of stable and secure housing for PEH. Previous research has illustrated the beneficial effects of housing for PEH on mental and physical health outcomes [87], as well as reducing non-routine health service usage [88]. PEH have also been demonstrated to have reduced access to primary health care and outpatient clinics [89,90], and as a result promoting and enhancing access to appropriate healthcare and social support for this group is important to appropriately manage frailty.

In clinical practice, the management of frailty in older people is often led by Geriatric Medicine, who provide robust functional assessment, alongside intensive input from allied health professionals such as occupational health and physiotherapy services. Given the level of frailty in PEH and issues around accelerated ageing in this group, a similar model of specialist integrated care is required for this group to better meet their holistic needs. Interventions may include exercise and nutrition [21], as well as practical housing strategies. Healthcare providers should complete a thorough assessment and modify interventions according to the feasibility in the specific environment in which a patient is living. For example, an adult living on the streets may find it difficult to follow a regular exercise regimen because of broken, stolen or unavailable exercise equipment, a lack of personal space, or a concern of being seen as a possible target [14]. Health professionals should be aware of these potential challenges and develop tailored interventions to address and mitigate them effectively.

Given the high rates of pre-frailty, future research would be beneficial to determine the impact of screening for this in PEH to determine if preventative care strategies could reduce the likelihood of frailty occurring. Appropriate management strategies need to be in place to manage frailty detected in PEH, and approaches to including these data in considerations of the social cost of homelessness, as it is important that there is a clear purpose to identifying frailty in PEH. Research is currently underway into developing frailty specific measures for PEH [91]. These may represent more appropriate instruments for detecting frailty compared to existing instruments that tend to focus on an older population. Models that also involve social and psychological domains, as well as physical issues, may be more helpful in assessing frailty in PEH. This may include social isolation, cognitive decline, addiction issues and environmental stressors (such as exposure to unstable living conditions and lack of access to adequate nutrition). Whilst similar to existing measures in capturing physical frailty, these tools would differ by integrating psychosocial and environmental dimensions and may be more helpful in identifying what supports are most required for frailty in PEH.

Previous studies on frailty interventions for PEH have illustrated the importance of addressing all domains of frailty including physical, psychological and social elements, and considering addiction issues when delivering these interventions [26,48].

This review also identified a paucity of research addressing risk factors of frailty in PEH apart from homelessness itself. Further research in this area would be helpful to highlight those most at risk of frailty and to better understand how being homeless contributes to frailty. This in turn may lead to more relevant strategies being developed to reverse frailty in PEH.

## 5 Conclusion

The results of this systematic review and meta-analysis have demonstrated high prevalence of frailty and pre-frailty among PEH, providing new insight into the needs of this vulnerable group and the need to develop effective interventions. This supports the existing evidence that PEH have increased rates of geriatric syndromes, multimorbidity and functional impairments [14,92], and experience premature ageing [14,93]. Limited literature was identified around risk factors for frailty in PEH, though possible determinants may include being female, increased years being homeless, and drug addiction. Screening for frailty in this group should be further examined in clinical practice and the development of bespoke and comprehensive services to optimise the care of frail PEH alongside sensible housing strategies is required. There is an opportunity for future research to develop an appropriate tool for measuring frailty in PEH and practical interventions to manage frailty and increase resilience in this population.

## Funding

Thomas Cronin has been supported by an ICGP (Irish College of General Practitioners) Aspire Fellowship and the Dr Jim Slein Research Bursary during this study.

## Ethical standards

As this is a review study and there were no human participants, ethical approval was not sought.

## CRediT authorship contribution statement

**Thomas Cronin:** Writing – review & editing, Writing – original draft, Project administration, Methodology, Investigation, Formal analysis, Data curation, Conceptualization. **David Healy:** Writing – review & editing, Validation, Investigation, Formal analysis. **Noel McCarthy:** Writing – review & editing, Supervision, Methodology, Formal analysis. **Susan M Smith:** Writing – review & editing, Supervision, Methodology, Formal analysis, Conceptualization. **John Travers:** Writing – review & editing, Supervision, Methodology, Formal analysis, Conceptualization.

## Declaration of competing interest

The authors declare that they have no known competing financial interests or personal relationships that could have appeared to influence the work reported in this paper.

## Appendix Supplementary materials

Image, application 1
