# Supplementary material for: Prevalence and risk factors of frailty in people experiencing homelessness: A systematic review and meta-analysis
Source: J Frailty Aging. 2025 Mar 4;14(2):100029. doi: 10.1016/j.tjfa.2025.100029 (PMC12184070; doi:10.1016/j.tjfa.2025.100029)
Supplement: Supplementary file 1 [file mmc1.docx]

| **Section and Topic** | **Item #** | **Checklist item** | **Location where item is reported** |
| --- | --- | --- | --- |
| **TITLE** | | |  |
| Title | 1 | Identify the report as a systematic review. | 1 |
| **ABSTRACT** | | |  |
| Abstract | 2 | See the PRISMA 2020 for Abstracts checklist. | 2 |
| **INTRODUCTION** | | |  |
| Rationale | 3 | Describe the rationale for the review in the context of existing knowledge. | 3 |
| Objectives | 4 | Provide an explicit statement of the objective(s) or question(s) the review addresses. | 4 |
| **METHODS** | | |  |
| Eligibility criteria | 5 | Specify the inclusion and exclusion criteria for the review and how studies were grouped for the syntheses. | 4-5 |
| Information sources | 6 | Specify all databases, registers, websites, organisations, reference lists and other sources searched or consulted to identify studies. Specify the date when each source was last searched or consulted. | 5 |
| Search strategy | 7 | Present the full search strategies for all databases, registers and websites, including any filters and limits used. | 5 |
| Selection process | 8 | Specify the methods used to decide whether a study met the inclusion criteria of the review, including how many reviewers screened each record and each report retrieved, whether they worked independently, and if applicable, details of automation tools used in the process. | 5-6 |
| Data collection process | 9 | Specify the methods used to collect data from reports, including how many reviewers collected data from each report, whether they worked independently, any processes for obtaining or confirming data from study investigators, and if applicable, details of automation tools used in the process. | 5-6 |
| Data items | 10a | List and define all outcomes for which data were sought. Specify whether all results that were compatible with each outcome domain in each study were sought (e.g. for all measures, time points, analyses), and if not, the methods used to decide which results to collect. | 5-6 |
|  | 10b | List and define all other variables for which data were sought (e.g. participant and intervention characteristics, funding sources). Describe any assumptions made about any missing or unclear information. | 5-6 |
| Study risk of bias assessment | 11 | Specify the methods used to assess risk of bias in the included studies, including details of the tool(s) used, how many reviewers assessed each study and whether they worked independently, and if applicable, details of automation tools used in the process. | 5 |
| Effect measures | 12 | Specify for each outcome the effect measure(s) (e.g. risk ratio, mean difference) used in the synthesis or presentation of results. | 6 |
| Synthesis methods | 13a | Describe the processes used to decide which studies were eligible for each synthesis (e.g. tabulating the study intervention characteristics and comparing against the planned groups for each synthesis (item #5)). | 5-6 |
|  | 13b | Describe any methods required to prepare the data for presentation or synthesis, such as handling of missing summary statistics, or data conversions. | 5-6 |
|  | 13c | Describe any methods used to tabulate or visually display results of individual studies and syntheses. | 5-6 |
|  | 13d | Describe any methods used to synthesize results and provide a rationale for the choice(s). If meta-analysis was performed, describe the model(s), method(s) to identify the presence and extent of statistical heterogeneity, and software package(s) used. | 5-6 |
|  | 13e | Describe any methods used to explore possible causes of heterogeneity among study results (e.g. subgroup analysis, meta-regression). | 5-6 |
|  | 13f | Describe any sensitivity analyses conducted to assess robustness of the synthesized results. | N/A |
| Reporting bias assessment | 14 | Describe any methods used to assess risk of bias due to missing results in a synthesis (arising from reporting biases). | N/A |
| Certainty assessment | 15 | Describe any methods used to assess certainty (or confidence) in the body of evidence for an outcome. | N/A |
| **RESULTS** | | |  |
| Study selection | 16a | Describe the results of the search and selection process, from the number of records identified in the search to the number of studies included in the review, ideally using a flow diagram. | Figure 1 |
|  | 16b | Cite studies that might appear to meet the inclusion criteria, but which were excluded, and explain why they were excluded. | Figure 1 |
| Study characteristics | 17 | Cite each included study and present its characteristics. | Supplementary material |
| Risk of bias in studies | 18 | Present assessments of risk of bias for each included study. | Supplementary material |
| Results of individual studies | 19 | For all outcomes, present, for each study: (a) summary statistics for each group (where appropriate) and (b) an effect estimate and its precision (e.g. confidence/credible interval), ideally using structured tables or plots. | 10 |
| Results of syntheses | 20a | For each synthesis, briefly summarise the characteristics and risk of bias among contributing studies. | Supplementary material |
|  | 20b | Present results of all statistical syntheses conducted. If meta-analysis was done, present for each the summary estimate and its precision (e.g. confidence/credible interval) and measures of statistical heterogeneity. If comparing groups, describe the direction of the effect. | Figure 3 /4 |
|  | 20c | Present results of all investigations of possible causes of heterogeneity among study results. | 10 |
|  | 20d | Present results of all sensitivity analyses conducted to assess the robustness of the synthesized results. | N/A |
| Reporting biases | 21 | Present assessments of risk of bias due to missing results (arising from reporting biases) for each synthesis assessed. | N/A |
| Certainty of evidence | 22 | Present assessments of certainty (or confidence) in the body of evidence for each outcome assessed. | N/A |
| **DISCUSSION** | | |  |
| Discussion | 23a | Provide a general interpretation of the results in the context of other evidence. | 11-12 |
|  | 23b | Discuss any limitations of the evidence included in the review. | 13-14 |
|  | 23c | Discuss any limitations of the review processes used. | 13-14 |
|  | 23d | Discuss implications of the results for practice, policy, and future research. | 14-15 |
| **OTHER INFORMATION** | | |  |
| Registration and protocol | 24a | Provide registration information for the review, including register name and registration number, or state that the review was not registered. | 4 |
|  | 24b | Indicate where the review protocol can be accessed, or state that a protocol was not prepared. | 4 |
|  | 24c | Describe and explain any amendments to information provided at registration or in the protocol. | 4 |
| Support | 25 | Describe sources of financial or non-financial support for the review, and the role of the funders or sponsors in the review. | 15 |
| Competing interests | 26 | Declare any competing interests of review authors. | 15 |
| Availability of data, code and other materials | 27 | Report which of the following are publicly available and where they can be found: template data collection forms; data extracted from included studies; data used for all analyses; analytic code; any other materials used in the review. | Supplementary material |

*From:*  Page MJ, McKenzie JE, Bossuyt PM, Boutron I, Hoffmann TC, Mulrow CD, et al. The PRISMA 2020 statement: an updated guideline for reporting systematic reviews. BMJ 2021;372:n71. doi: 10.1136/bmj.n71

**Supplementary Material Section 2 – Search strategies**

**Ovid MEDLINE search strategy**


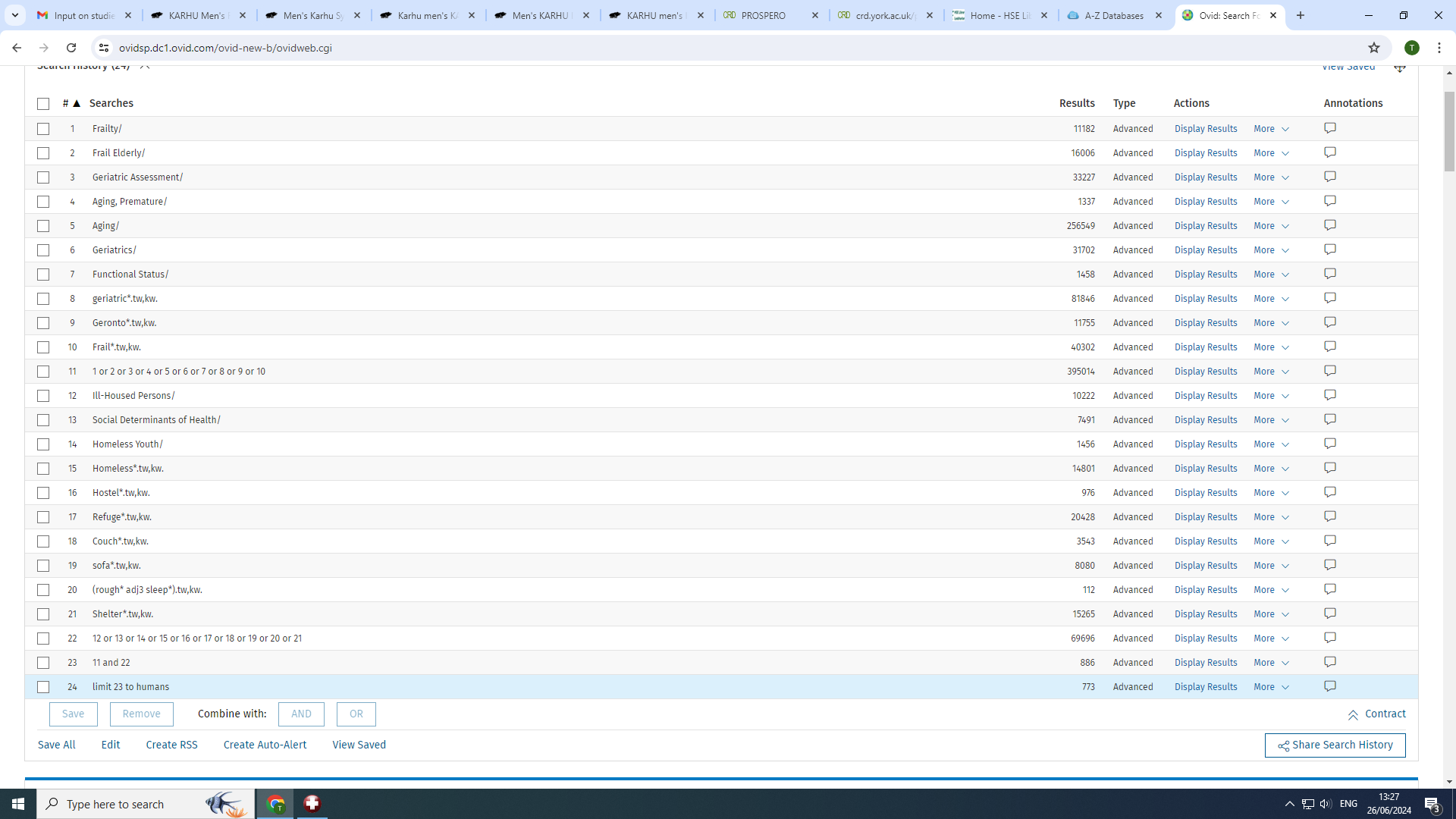


**PsycInfo search strategy**


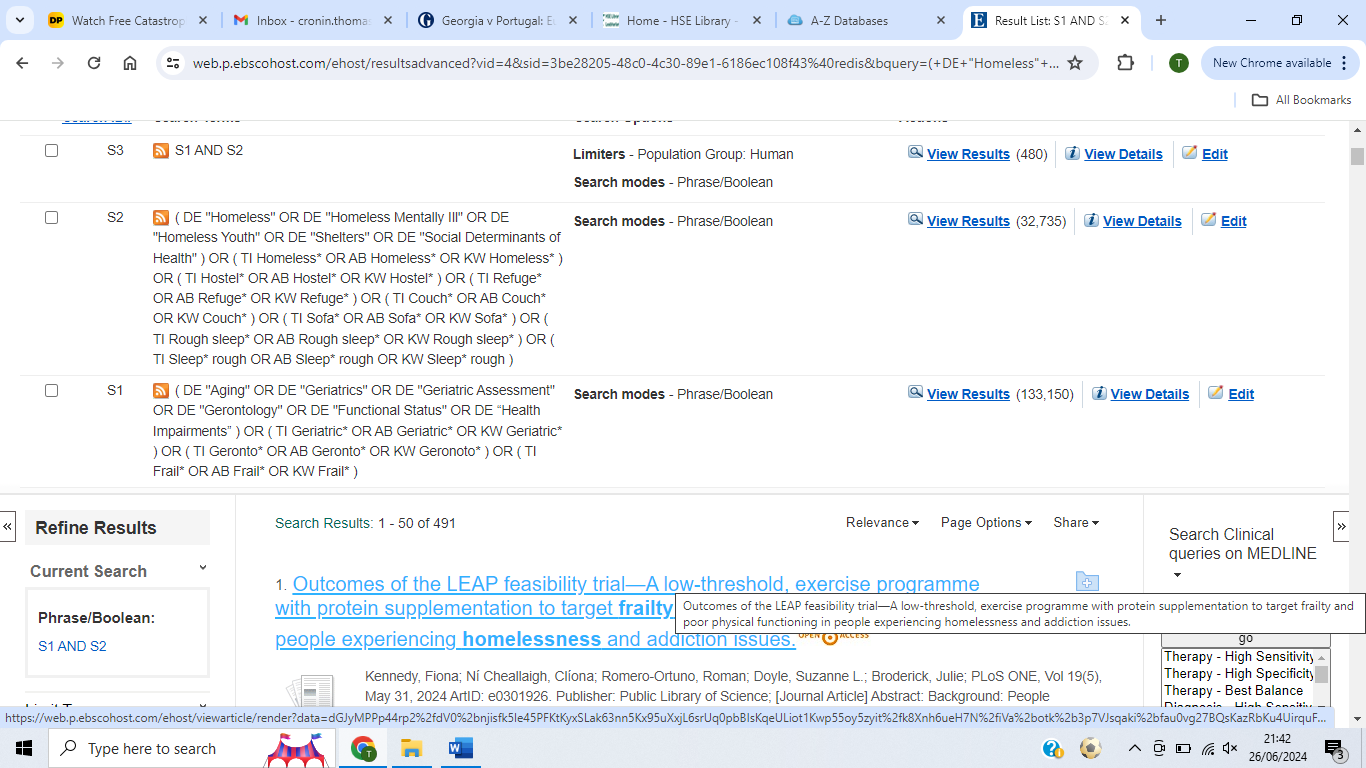


**Web of Science search strategy**


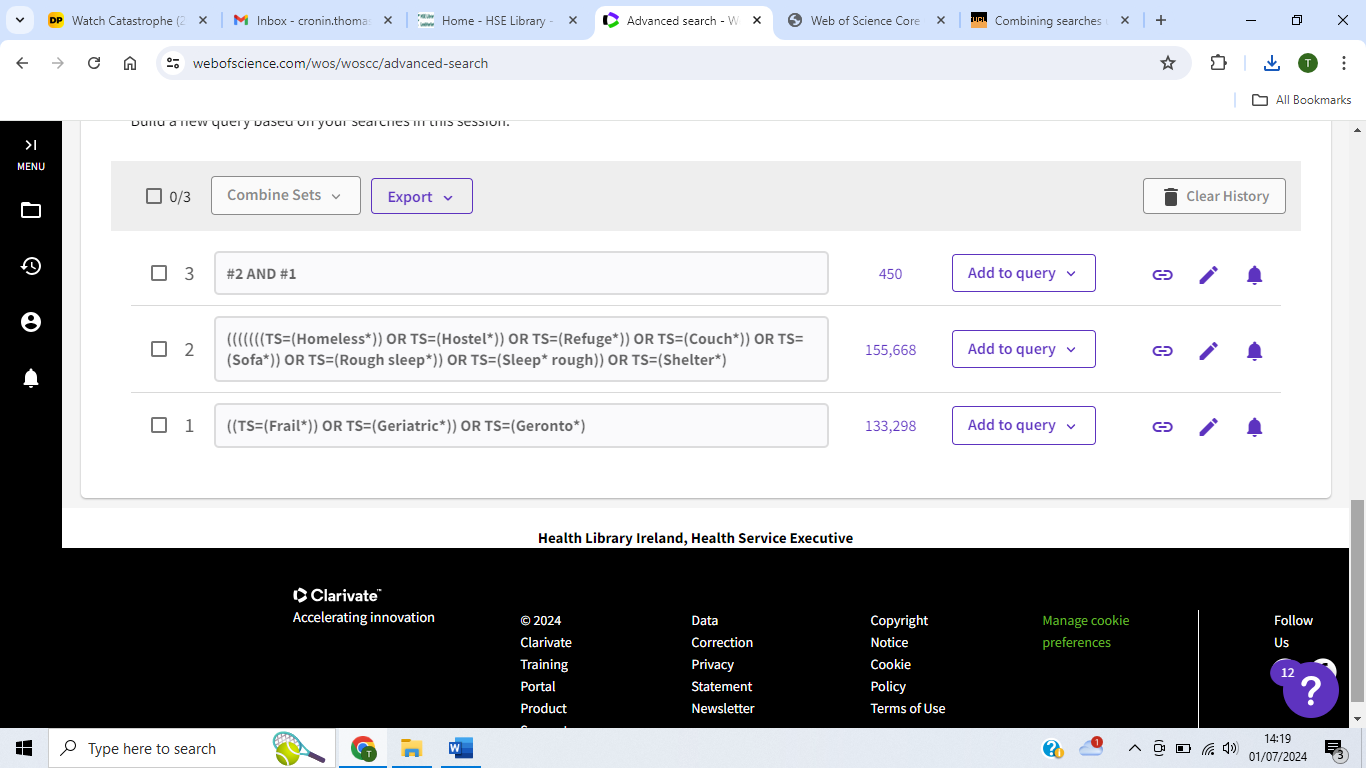


**CINAHL Complete Search Strategy**


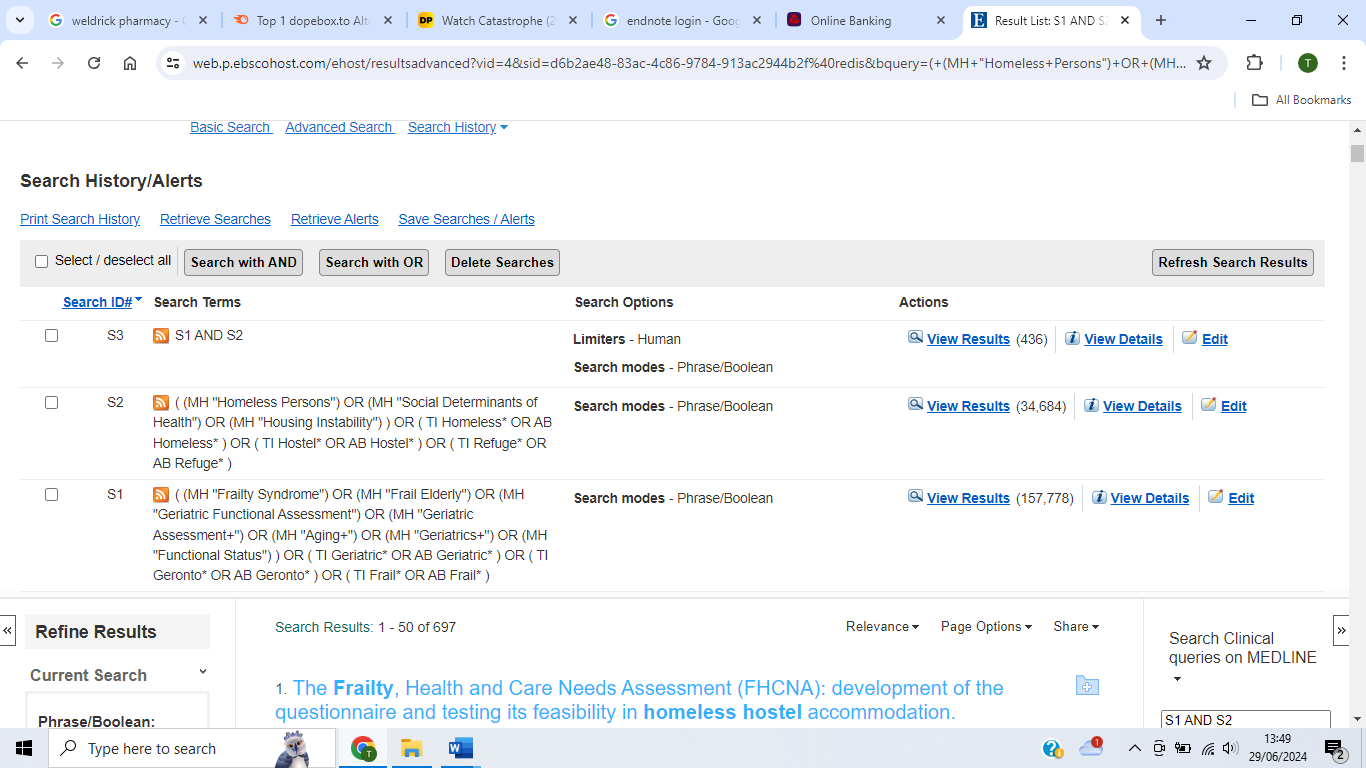


**Supplementary Material Section 3 – Data extraction table**

| **First author and year of publication** | **Study location** | **Study type** | **Aim** | **FEANSTA Operational Category** | **Recruitment strategy / Exclusion criteria** | **Sample size** | **Participant characteristics** | **Frailty measure used** | **Frailty prevalence** | **Frailty risk factors (biological, lifestyle, psychological and social)** |
| --- | --- | --- | --- | --- | --- | --- | --- | --- | --- | --- |
| Brown, 2013 [1] | USA | Cross-sectional | To identify factors associated with the presence of geriatric syndromes in a sample of older homeless adults. | People living rough, People in emergency accommodation, People in accommodation for the homeless | Systematic random sampling in day shelters and homeless hostels, stratified at a 4:1 ratio of men to women to reflect the homeless population in Boston area.  Exclusion: < 50 years, unable to communicate in English, altered consciousness level. | N=250 | Average (mean) age = 56  19% (n=48) were female | Fried criteria | 16% (n=40) | *Biological*  Of the participants recorded as having frailty, 95% (n=38) had >1 other geriatric syndrome, 88% (n=35) had >2 other geriatric syndromes, and 38% (n=15) had >4 other geriatric syndromes. |
| Salem, 2013 [2] | USA | Cross-sectional | To determine factors associated with frailty in people who are homeless | People living rough, People in emergency accommodation, People in accommodation for the homeless | Voluntary response sampling from day shelters and homeless hostel.  Exclusion: age < 40 years, unable to communicate in English, altered level of consciousness. | N=150 | Average (mean) = age 52    50% (n=75) were female. | Frailty Index | 54% (n=81) | *Biological*  Using stepwise regression increased age, lower nutritional status (measure by mini-nutritional assessment) and being female were significantly associated with being frail in the studied population.  *Social*  Increased use of healthcare was also associated with being frail. |
| Salem, 2019 [3] | USA | Cross-sectional | To explore factors associated with frailty among formerly incarcerated, currently homeless women enrolled in a randomised control trial. | People living rough, People in emergency accommodation, People in accommodation for the homeless | Voluntary response sampling from day shelters.  Exclusion: > 65 years, not previously incarcerated, no prior drug use. | N=130 | Average (mean) age = 39  100% (n=130) were female. | Tilburg Frailty Index | Not recorded | *Biological*  Using multivariable regression, those who were older had higher physical frailty scores (*p*<0.01), whilst lower levels of psychological frailty (*p*<0.05).  *Lifestyle*  Those who had a higher drug dependence had greater physical, psychological and social frailty scores (*p*<0.05).  *Psychological*  Higher PTSD symptom scores had higher levels of physical frailty (*p*<0.05)  *Social*  Those who had a higher number of years homeless had higher physical and psychological frailty scores (*p*<0.05). |
| Rogans-Watson, 2020 [4] | UK | Cross-sectional | To determine the extent of frailty and geriatric conditions in people living in a homeless hostel. | People in accommodation for the homeless | Convenience sampling from a homeless hostel.  Exclusion: significant communication barrier. | N=33 | Average (mean) age = 56  9% (n=3) were female. | Fried Criteria, Edmonton Frail Scale, Clinical Frailty Scale | Fried Criteria:  39% (n=13) were pre-frail and 55% (n=18) were frail.  Edmonton Frail Scale: classified 18% (n=6) as pre-frail and 55% (n=18) as frail.  Clinical Frailty Scale: 33% (n=11) were pre-frail and 48% (n=16) were frail. | Nil applicable |
| Kiernan, 2021 [5] | Ireland | Cross-sectional | To profile adult in-patients registered as homeless using a broad battery of physical performance and functional limitation tests. | People living rough, People in emergency accommodation, People in accommodation for the homeless | Convenience sampling from inpatients flagged as homeless in a large acute teaching hospital.  Exclusion: inability to communicate in English, altered consciousness level, serious medical or orthopaedic issue, confirmed pregnancy. | N=63 | Average (median) age = 45  32% (n=21) were female. | Clinical Frailty Scale | 29% (n=18) were pre-frail and 40% (n=25) were frail | *Biological*  The distribution of frailty scores was higher in females than males (*p*<0.05). No difference in frailty scores was observed between age groups. |
| Nyamathi, 2022 [6] | USA | Single-arm study | To assess the impact of a nurse-led intervention on reducing substance use amongst people experiencing homelessness | People living rough, People in emergency accommodation, People in accommodation for the homeless | Voluntary response sampling from day shelters and homeless hostel.  Exclusion: active TB or treatment for latent TB, serum aspartate aminotransferase (AST) level of 5 times the upper limit of normal, HIV-infected, pregnant, altered level of consciousness, no current or recent substance use within the last 12 months | N=50 | Average (mean) age = 54  26% (n=13) were female. | Tilburg Frailty Index | 46% (n=23) | Nil applicable |
| Lowrie 2023, [7] | UK | Cross-sectional | To report on baseline findings of a randomised controlled trial involving people experiencing homelessness. | People living rough, People in emergency accommodation, People in accommodation for the homeless | Purposive sampling through staff working in homeless accommodations identifying and approaching potential participants who had overdosed in the preceding 6 months and voluntary response sampling from day shelters.  Exclusion: living in a residential or community-based rehabilitation facility that has direct access in-house medical care, no drug overdose in the preceding six months. | N=71 | Not available | Fried Criteria | 28% (n=20) were pre-frail and 70% (n=50) were frail. | Nil applicable |
| Speck, 2023 [8] | Germany | Cross-sectional | To collect data on the prevalence rates of physical frailty in a social services charity that provides healthcare for homeless people. | People living rough, People in emergency accommodation, People in accommodation for the homeless | Convenience sampling of from a clinic for people experiencing homelessness.  Exclusion: inability to communicate in German, English, Romanian, or Bulgarian, fracture of lower limbs / wheelchair dependent, altered level of consciousness. | N=60 | Average (median) age = 48  12% (n=7) were female. | Fried Criteria | 62% (n=36) were pre-frail and 22% (n=13) were frail. | Nil applicable |
| Nyamathi, 2023 [9] | USA | Case-control | To assess the presence of antibodies against SARS-CoV-2 among people who are homeless and associations with key variables. | People living rough, People in emergency accommodation, People in accommodation for the homeless | Convenience sample from homeless accommodation and drug treatment sites.  Exclusion: inability to speak English or Spanish, no current or recent substance use within the last 12 months. | N=105 | Average (mean) age = 45  % of female was not available. | Tilburg Frailty Indicator | 18% (n=19) | Nil applicable |
| Shulman, 2024 [10] | UK | Feasibility | To co-develop a Frailty, Health and Care Needs Assessment (FHCNA) in partnership with inclusion health and non-clinical homelessness staff | People in accommodation for the homeless | Convenience sampling from a homeless hostel.  Exclusion: communication barrier. | N=74 | Average (mean) age = 49  27% (n=20) were female | Edmonton Frail Scale | 23% (n=17) were pre-frail and 53% (n=39) were frail. | Nil applicable |
| Kennedy 2024 [11] | Ireland | Feasibility | To explore the feasibility of an exercise and protein supplement to improve frailty in people experiencing homelessness. | People living rough, People in emergency accommodation, People in accommodation for the homeless | Voluntary response sampling from a day shelter.  Exclusion: altered conscious level, pregnancy, major physical impairments. | 31 | Average (mean) age = 45.  36% (n=11) were female. | Clinical Frailty Scale,  SHARE-FI | Clinical Frailty Scale: 26% (n=8) were pre-frail, and 23% (n=7) were frail. SHARE-FI: 19% (n=6) were pre-frail, and 16% (n=5) were frail. | Nil applicable |

References

1. Brown RT, Kiely DK, Bharel M, Mitchell SL. Factors associated with geriatric syndromes in older homeless adults. Journal of health care for the poor and underserved. 2013 May;24(2).
2. Salem BE, Nyamathi AM, Brecht ML, Phillips LR, Mentes JC, Sarkisian C, Leake B. Correlates of frailty among homeless adults. Western Journal of Nursing Research. 2013 Oct;35(9):1128-52.
3. Salem BE, Brecht ML, Ekstrand ML, Faucette M, Nyamathi AM. Correlates of physical, psychological, and social frailty among formerly incarcerated, homeless women. Health care for women international. 2019 Aug 20;40(7-9):788-812.
4. Rogans-Watson R, Shulman C, Lewer D, Armstrong M, Hudson B. Premature frailty, geriatric conditions and multimorbidity among people experiencing homelessness: a cross-sectional observational study in a London hostel. Housing, Care and Support. 2020 Sep 25;23(3/4):77-91.
5. Kiernan S, Ní Cheallaigh C, Murphy N, Dowds J, Broderick J. Markedly poor physical functioning status of people experiencing homelessness admitted to an acute hospital setting. Scientific Reports. 2021 May 10;11(1):9911.
6. Nyamathi A, Morisky D, Wall SA, Yadav K, Shin S, Hall E, Chang AH, White K, Arce N, Parsa T, Salem BE. Nurse‐led intervention to decrease drug use among LTBI positive homeless adults. Public Health Nursing. 2022 Jul;39(4):778-87.
7. Lowrie R, McPherson A, Mair FS, Stock K, Jones C, Maguire D, Paudyal V, Duncan C, Blair B, Lombard C, Ross S. Baseline characteristics of people experiencing homelessness with a recent drug overdose in the PHOENIx pilot randomised controlled trial. Harm reduction journal. 2023 Apr 4;20(1):46.
8. Speck C, Amberg I, Deichmann A, Keil L, Pauer M, Kolbe C, Schrader K. Frailty of Homeless People: Partial Publication of the Faho Study. Deutsches Ärzteblatt International. 2023 Oct;120(40):677.
9. Nyamathi A, Shin SS, Doratt BM, Jones‐Patten A, Salem B, Gelberg L, Lee D, Garfin D, Yadav K, Chang AH, White K. Correlates of SARS‐CoV‐2 anti‐RBD IgG antibody titers among persons experiencing homelessness in Los Angeles. Public Health Nursing. 2023 May;40(3):417-27.
10. Shulman C, Rogans-Watson R, Palipane N, Lewer D, Yeung M, Hudson BF. The Frailty, Health and Care Needs Assessment (FHCNA): development of the questionnaire and testing its feasibility in homeless hostel accommodation. Housing, Care and Support. 2024(ahead-of-print).
11. Kennedy F, Ní Cheallaigh C, Romero-Ortuno R, Doyle SL, Broderick J. Outcomes of the LEAP feasibility trial—A low-threshold, exercise programme with protein supplementation to target frailty and poor physical functioning in people experiencing homelessness and addiction issues. Plos one. 2024 May 31;19(5):e0301926.

**Supplementary Material Section 4 – JBI Critical Appraisal Instrument for Studies Reporting Prevalence Data**

| **First author and year of publication** | **Was the sample frame appropriate to address the target population?** | **Were study participants sampled in an appropriate way?** | **Was the sample size adequate?** | **Were the study subjects and the setting described in detail?** | **Was the data analysis conducted with sufficient coverage of the identified sample?** | **Were valid methods used for the identification of the condition?** | **Was the condition measured in a standard, reliable way for all participants?** | **Was there appropriate statistical analysis?** | **Was the response rate adequate, and if not, was the low response rate managed appropriately?** | **Proportion of Yeses** |
| --- | --- | --- | --- | --- | --- | --- | --- | --- | --- | --- |
| Brown, 2013 [1] | Yes | Yes | Yes | Yes | Unclear | Yes | Yes | Yes | Unclear | 78% (n=7) |
| Salem, 2013 [2] | Yes | Unclear | Yes | Yes | Unclear | Yes | Yes | Yes | Unclear | 67% (n=6) |
| Rogans-Watson, 2020 [3] | Unclear | Yes | No | Yes | Yes | Yes | Yes | Yes | Yes | 78% (n=7) |
| Kiernan, 2021 [4] | Yes | Yes | Unclear | Yes | Yes | Yes | Yes | Yes | Unclear | 78% (n=7) |
| Nyamathi, 2022 [5] | Yes | Unclear | Yes | Yes | Yes | Yes | Yes | Yes | Unclear | 78% (n=7) |
| Lowrie, 2023 [6] | Yes | Yes | Yes | Yes | Yes | Yes | Yes | Yes | Unclear | 89% (n=8) |
| Speck, 2023 [7] | Unclear | Unclear | Unclear | Yes | Yes | Yes | Yes | Yes | Yes | 67% (n=6) |
| Nyamathi, 2023 [8] | Unclear | Unclear | Unclear | Yes | Yes | Yes | Yes | Yes | Unclear | 56% (n=5) |
| Shulman, 2024 [9] | Unclear | Yes | Unclear | Unclear | Yes | Yes | Yes | Yes | Yes | 67% (n=6) |
| Kennedy, 2024 [10] | Unclear | Yes | No | Yes | Yes | Yes | Yes | Yes | Unclear | 67% (n=6) |

**References**

1. Brown RT, Kiely DK, Bharel M, Mitchell SL. Factors associated with geriatric syndromes in older homeless adults. Journal of health care for the poor and underserved. 2013 May;24(2).
2. Salem BE, Nyamathi AM, Brecht ML, Phillips LR, Mentes JC, Sarkisian C, Leake B. Correlates of frailty among homeless adults. Western Journal of Nursing Research. 2013 Oct;35(9):1128-52.
3. Rogans-Watson R, Shulman C, Lewer D, Armstrong M, Hudson B. Premature frailty, geriatric conditions and multimorbidity among people experiencing homelessness: a cross-sectional observational study in a London hostel. Housing, Care and Support. 2020 Sep 25;23(3/4):77-91.
4. Kiernan S, Ní Cheallaigh C, Murphy N, Dowds J, Broderick J. Markedly poor physical functioning status of people experiencing homelessness admitted to an acute hospital setting. Scientific Reports. 2021 May 10;11(1):9911.
5. Nyamathi A, Morisky D, Wall SA, Yadav K, Shin S, Hall E, Chang AH, White K, Arce N, Parsa T, Salem BE. Nurse‐led intervention to decrease drug use among LTBI positive homeless adults. Public Health Nursing. 2022 Jul;39(4):778-87.
6. Lowrie R, McPherson A, Mair FS, Stock K, Jones C, Maguire D, Paudyal V, Duncan C, Blair B, Lombard C, Ross S. Baseline characteristics of people experiencing homelessness with a recent drug overdose in the PHOENIx pilot randomised controlled trial. Harm reduction journal. 2023 Apr 4;20(1):46.
7. Speck C, Amberg I, Deichmann A, Keil L, Pauer M, Kolbe C, Schrader K. Frailty of Homeless People: Partial Publication of the Faho Study. Deutsches Ärzteblatt International. 2023 Oct;120(40):677.
8. Nyamathi A, Shin SS, Doratt BM, Jones‐Patten A, Salem B, Gelberg L, Lee D, Garfin D, Yadav K, Chang AH, White K. Correlates of SARS‐CoV‐2 anti‐RBD IgG antibody titers among persons experiencing homelessness in Los Angeles. Public Health Nursing. 2023 May;40(3):417-27.
9. Shulman C, Rogans-Watson R, Palipane N, Lewer D, Yeung M, Hudson BF. The Frailty, Health and Care Needs Assessment (FHCNA): development of the questionnaire and testing its feasibility in homeless hostel accommodation. Housing, Care and Support. 2024(ahead-of-print).
10. Kennedy F, Ní Cheallaigh C, Romero-Ortuno R, Doyle SL, Broderick J. Outcomes of the LEAP feasibility trial—A low-threshold, exercise programme with protein supplementation to target frailty and poor physical functioning in people experiencing homelessness and addiction issues. Plos one. 2024 May 31;19(5):e0301926.

**Supplementary Material Section 5 – JBI Critical appraisal checklist for analytical cross-sectional studies**

| **First author and year of publication** | **Were the criteria for inclusion in the sample clearly defined?** | **Were the study subjects and the setting described in detail?** | **Was the exposure measured in a valid and reliable way?** | **Were objective, standard criteria used for measurement of the condition?** | **Were confounding factors identified?** | **Were strategies to deal with confounding factors stated?** | **Were the outcomes measured in a valid and reliable way?** | **Was appropriate statistical analysis used?** | **Proportion of Yeses** |
| --- | --- | --- | --- | --- | --- | --- | --- | --- | --- |
| Brown, 2013 [1] | Yes | Yes | No | Yes | No | Yes | Yes | Yes | 75% (n=6) |
| Salem, 2013 [2] | Unclear | Yes | No | Yes | No | No | Yes | Yes | 50% (n=4) |
| Salem, 2019 [3] | Unclear | Yes | No | Yes | No | No | Yes | Yes | 50% (n=4) |
| Kiernan, 2021 [4] | Yes | Yes | No | Yes | No | No | Yes | Yes | 75% (n=6) |

**References**

1. Brown RT, Kiely DK, Bharel M, Mitchell SL. Factors associated with geriatric syndromes in older homeless adults. Journal of health care for the poor and underserved. 2013 May;24(2).
2. Salem BE, Nyamathi AM, Brecht ML, Phillips LR, Mentes JC, Sarkisian C, Leake B. Correlates of frailty among homeless adults. Western Journal of Nursing Research. 2013 Oct;35(9):1128-52.
3. Salem BE, Brecht ML, Ekstrand ML, Faucette M, Nyamathi AM. Correlates of physical, psychological, and social frailty among formerly incarcerated, homeless women. Health care for women international. 2019 Aug 20;40(7-9):788-812.
4. Kiernan S, Ní Cheallaigh C, Murphy N, Dowds J, Broderick J. Markedly poor physical functioning status of people experiencing homelessness admitted to an acute hospital setting. Scientific Reports. 2021 May 10;11(1):9911.
